# How consistent are lordosis, range of movement and lumbo-pelvic rhythm in people with and without back pain?

**DOI:** 10.1186/s12891-016-1250-1

**Published:** 2016-09-22

**Authors:** Robert A. Laird, Peter Kent, Jennifer L. Keating

**Affiliations:** 1 Department of Physiotherapy, Monash University, PO Box 527, Frankston, VIC 3199 Australia; 2 School of Physiotherapy and Exercise Science, Curtin University, Perth, Australia; 3 Department of Sports Science and Clinical Biomechanics, University of Southern Denmark, Odense, Denmark; 4 380 Springvale Rd, Forest Hill, 3131 Melbourne, VIC Australia

**Keywords:** Low back pain, Movement disorders, Posture, ROM, Lordosis, Lumbo-pelvic rhythm, Reliability

## Abstract

**Background:**

Comparing movements/postures in people with and without lower back pain (LBP) may assist identifying LBP-specific dysfunction and its relationship to pain or activity limitation. This study compared the consistency in lumbo-pelvic posture and movement (range and pattern) in people with and without chronic LBP (>12 week’s duration).

**Methods:**

Wireless, wearable, inertial measurement units measured lumbar lordosis angle, range of movement (ROM) and lumbo-pelvic rhythm in adults (*n* = 63). Measurements were taken on three separate occasions: two tests on the same day with different raters and a third (intra-rater) test one to two weeks later. Participants performed five repetitions of tested postures or movements. Test data were captured automatically. Minimal detectable change scores (MDC_90_) provided estimates of between-test consistency.

**Results:**

There was no significant difference between participants with and without LBP for lordosis angle. There were significant differences for pelvic flexion ROM (LBP 60.8°, NoLBP 54.8°, F(1,63) = 4.31, *p* = 0.04), lumbar right lateral flexion ROM (LBP 22.2°, NoLBP 24.6° F(1,63) = 4.48, *p* = .04), trunk right lateral flexion ROM (LBP 28.4°, NoLBP 31.7°, F(1,63) = 5.9, *p* = .02) and lumbar contribution to lumbo-pelvic rhythm in the LBP group (LBP 45.8 %, F(1,63) = 4.20, NoLBP 51.3 % *p* = .044). MDC_90_ estimates for intra and inter-rater comparisons were 10°–15° for lumbar lordosis, and 5°–15° for most ROM. For lumbo-pelvic rhythm, we found 8–15 % variation in lumbar contribution to flexion and lateral flexion and 36–56 % variation in extension. Good to excellent agreement (reliability) was seen between raters (mean *r* = .88, ICC (2,2)).

**Conclusion:**

Comparisons of ROM between people with and without LBP showed few differences between groups, with reduced relative lumbar contribution to trunk flexion. There was no difference between groups for lordosis. Wide, within-group differences were seen for both groups for ROM and lordosis. Due to variability between test occasions, changes would need to exceed 10°–15° for lumbar lordosis, 5°–15° for ROM components, and 8–15 % of lumbar contribution to lumbo-pelvic rhythm, to have 90 % confidence that movements had actually changed. Lordosis, range of movement and lumbo-pelvic rhythm typically demonstrate variability between same-day and different-day tests. This variability needs to be considered when interpreting posture and movement changes.

**Electronic supplementary material:**

The online version of this article (doi:10.1186/s12891-016-1250-1) contains supplementary material, which is available to authorized users.

## Background

In a recent ‘Global Burden of Disease Study’ [1], low back pain (LBP) was rated as the health condition responsible for the most years lived with disability when all common diseases were considered. Despite considerable research efforts, it is still unclear why some people recover from LBP pain and others do not, or how to match available interventions to care-seekers [2]. Many studies have focused on movement irregularities and patterns in LBP. Movement range has been used to monitor recovery status following interventions, and various patterns of movement have been investigated, including lumbar versus pelvic (hip) contribution to trunk movement (often called lumbo-pelvic rhythm) [3–9]. Opinions vary regarding the utility of measuring movement range and patterns. Nevertheless, many non-invasive interventions continue to target movement dysfunction in people with LBP.

A concept with current support is that individuals have consistent, and therefore recognisable, patterns of posture and movement, which may contribute to ongoing LBP [10–13]. Movement patterns such as excessive end range lumbar movements or postures [14], excessive or reduced lumbar contribution to trunk flexion [15], trunk rigidity [16], loss of flexion relaxation response [17], and reduced proprioception [18, 19], amongst others, have been linked to LBP. Recent research supports the concept that individualised approaches to modification of posture and movement patterns might reduce LBP [20, 21]. However, the relationship between specific movement characteristics/postures and LBP remains unclear. A recent systematic review of common movement characteristics in people with and without LBP concluded that people with LBP typically have reduced range of lumbar spine movement, move more slowly and have reduced proprioception compared to people without LBP [22]. Another recent review found only limited evidence for identifying and monitoring changes to movement patterns or postures [23].

Aberrant movement (range or patterns) and/or postures associated with LBP might be identifiable, provided these movements were consistent and could be accurately measured. Of particular clinical interest is the consistency of an individual’s typical movement over short time periods (e.g., within a clinical session on the same day) and over longer time periods (e.g., 1 to 2weeks apart). Common therapeutic targets of ‘improving posture’ and normalising dysfunctional movements are often influenced by within-session or between-session changes in movements following a treatment. Therefore, knowledge of the kinematic stability of movement patterns both within and between treatment sessions is important to clinicians who aim to identify, label and treat movement ‘dysfunctions’. If movement/postural patterns normally fluctuate, and the variance in measures of movement/posture can be quantified, measurements outside the range of expected variation are likely to represent true movement alteration/adaptation. Those adaptive movements could be used to quantify response to treatment or to identify movements that either trigger, or are a response to, LBP.

Investigating the associations between movement and pain has been limited by difficulty in measuring and monitoring typical movement/posture both within clinical settings and in normal daily activity. Technological advances with movement sensors have enabled new opportunities to investigate the relationship between movement and pain [24–26]. These devices are skin surface-mounted and generate data on lumbo-pelvic movements and postures, such as angle, timing, position and concurrent surface electromyography. There is preliminary evidence of high levels of accuracy relative to laboratory based opto-electronic measurement systems and they appear to have sufficient accuracy for clinical applications [24, 27].

This study investigated and compared consistency in lumbo-pelvic posture and movement (range and pattern) in people with and without chronic LBP (>12 week’s duration). We examined the consistency (repeatability/measurement stability) of three types of lumbo-pelvic kinematic characteristics: (i) the postural characteristic of lordosis, (ii) range of movement (ROM) of flexion, extension, and lateral flexion, and (iii) lumbar compared to pelvic contributions to movement (lumbo-pelvic rhythm). Three types of movement consistency were of interest: 1) the consistency demonstrated when an individual repeats the same movement within a single test, 2) the consistency demonstrated when a person is tested twice by two different raters on the same day, and 3) the consistency demonstrated when a person has a repeated test by the same person 7–14 days after the first test.

## Methods

### Study selection: inclusion and exclusion criteria

Participants (with and without LBP) were recruited by poster and word-of-mouth advertising from private physiotherapy clinics and a university. People with LBP (LBP group) were included if they had back +/− leg pain for > 12 weeks and a pain score of > 2 on a 0 to 10 Numerical Rating Scale (average of worst, current, usual pain intensity) [28]. Exclusion criteria were any of the following: (i) previous lumbar surgery, (ii) any invasive spinal procedures for LBP, including therapeutic injections, within the last 12 months, (iii) pregnancy (iv) neoplasm, infection, fracture, inflammatory disease, neurological disease or any metabolic disorder that had the potential to affect the lumbo-pelvic region, (v) implanted electrical medical device, (vi) any medical abnormalities or conditions (e.g., knee or hip conditions) that in the opinion of the clinician would substantively interfere with an ability to participate in the study, (vii) a known allergic skin reaction to adhesive tapes or plasters, or (viii) BMI > 30 (where it becomes difficult to palpate bony landmarks). Participants recruited into the sample without back pain (NoLBP group) were excluded if they had (i) back pain at the time of testing, (ii) an episode of back pain that had necessitated attending a medical practitioner or allied health professional in the last 12 months, (iii) time off work due to back pain in the last 12 months or, (iv) any back pain during or between testing procedures. All potential participants were screened for suitability by a trained administrator, by direct contact and follow-up phone call if clarification was required, and then invited to participate. Ethics approval was obtained from Monash University (approval numberCF12/1995-20 12001090). All participants gave written informed consent.

### Measurement protocol

**Fig. 1:**
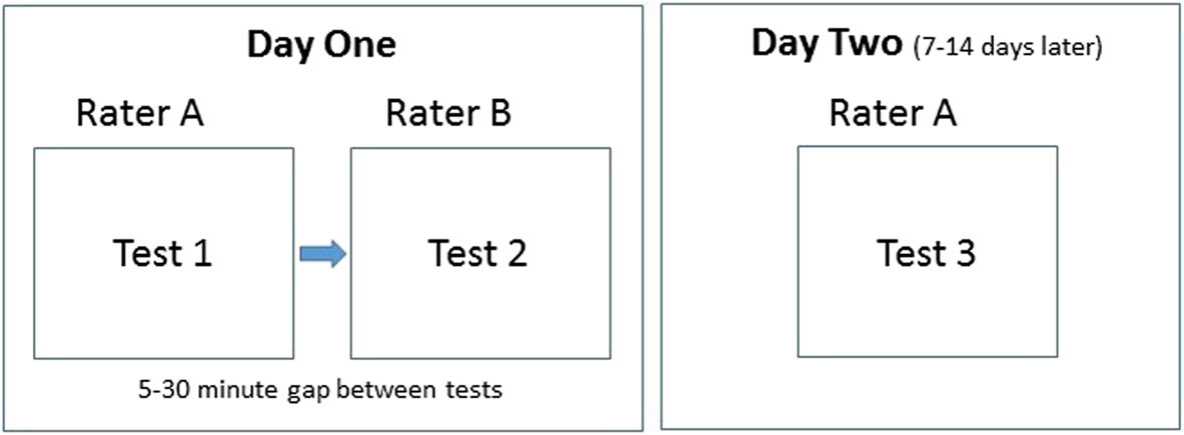
Flow diagram of assessment procedures

Figure 1 presents the test procedures. Each participant was tested on two separate days. On the first test day, they were tested twice (Test 1 and Test 2) by two different raters (Raters A and B). On the second test day, they were assessed once (Test 3) by Rater A. On each test occasion, participants were assessed while they performed five repetitions of each movement. Data were collected at two geographic locations by physiotherapists with a minimum of 2 years’ clinical experience.

To standardise the testing procedures, 3 h of practice for standardised palpation of bony landmarks, sensor placement and measurement procedures preceded the initial data collection. Standardised instructions were used by both raters with pre-determined verbal cues for each movement test. Rater order (i.e., who administered Tests 1 or 2) was randomised pragmatically by rater availability. Participants were tested in the same room for all tests, and where possible, were tested at a similar time of day.

All kinematic data were automatically captured by the ViMove system independently of actions by the rater.

### Equipment

The ViMove system (DorsaVi, Australia) is an inertial measurement system comprised of two wireless movement sensors containing a triaxial accelerometer, a triaxial gyroscope and a magnetometer, two wireless surface electromyography (EMG) sensors (these EMG data were not reported in this paper), and a small wireless recording device that can be easily carried (e.g., in a pocket). The manufacturer reports average differences of < 1° for single plane, through-range movements when comparing matched measurements from the ViMove and a Fastrak opto-electronic device [29]. The ViMove movement sensors collect data at approximately 20 Hz.

### Test procedures

**Fig. 2:**
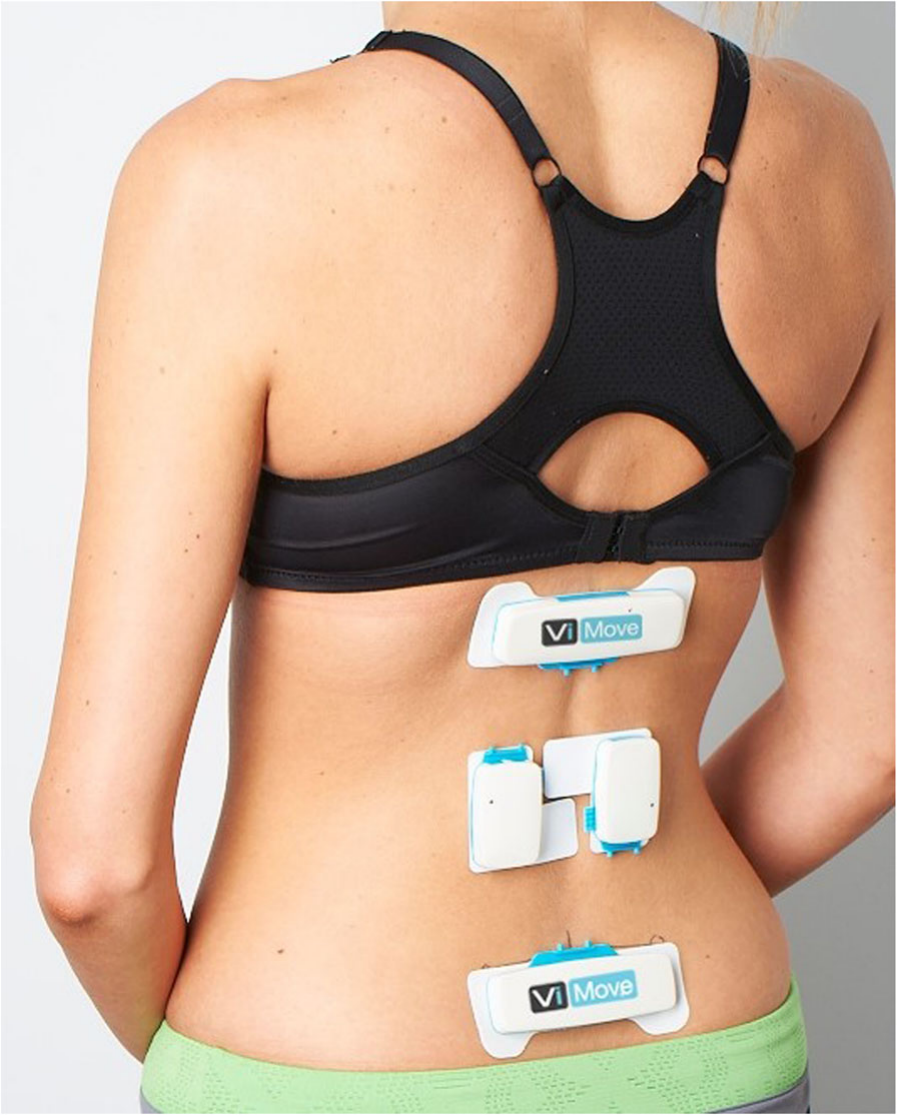
Placement of the ViMove sensors

Participants were partially undressed to expose the body from T12 to the posterior superior iliac spines (PSIS) (see Fig. 2). Shoes were removed. The upper border of each PSIS was palpated and marked by Rater 1. To standardise sensor placement, the distance from the PSIS marker to the floor was recorded using a rigid vertical ruler and right-angled square. These measurements were used to replicate PSIS markings in subsequent testing [30]. A plastic template (part of the ViMove system) for standardising relative sensor placement was then aligned to the marking on the PSIS and used to guide sensor attachments. Movement sensors were attached to the skin over the T12 and S2 spinous processes using disposable adhesive pads. Movements were then demonstrated by the rater, after which participants were instructed to move through a standardised sequence of movements (summarised in Additional file 1).

During these movements, data on lumbo-pelvic angles and ROM were recorded automatically by the device. The only role of the rater was to request the required movement in the required sequence and initiate the data collection process. On completion of a test, sensors and adhesive pads were removed and the skin was wiped clean. Participants rested for 5 min then the entire procedure was immediately repeated by a second rater. Each rater was blind to data collected by the other rater with the exception of the measurement of the vertical distance of the PSIS from the floor. Participants then returned 7–14 days later for a repeat assessment (Test 3) by Rater A. For participants with LBP, pain was recorded using three Numerical Rating Scales (worst pain =10, no pain =0), and the average of current, usual and worst pain over the previous 2 weeks was used [31]). Activity limitation was assessed using the Roland Morris Disability Questionnaire [32]. Pain and activity limitation were recorded on both assessment occasions.

### Sample size

No existing data were available to inform sample size estimates. A sample of 60 adults aged 18–60 years (*n* = 30 with LBP, *n* = 30 without LBP) were recruited. This sample size would allow detection of a correlation of 0.44 or more between repeated measures in each group of 30, with an alpha of 0.05 and power of 0.8 [33]. Arbitrarily, we assumed this was an adequate threshold, as movement consistency that resulted in lower retest correlations would provide adequate evidence that the individual variations in movement patterns would be so large that patterns of movements would be too variable to be clinically interpretable. In addition, a sample size of 30 is recommended where researchers are studying differences between two sets of scores, as difference scores for samples of 30 or more are likely to assume a normal distribution and thereby provide more adequate data for parametric tests.

### Data analysis

Data on body position were sampled and recorded at approximately 20Hz for each of the five repetitions of flexion, extension and left and right lateral flexion movements. Averaged lumbar lordosis angle was recorded in standing over a 5-s period.

**Fig. 3:**
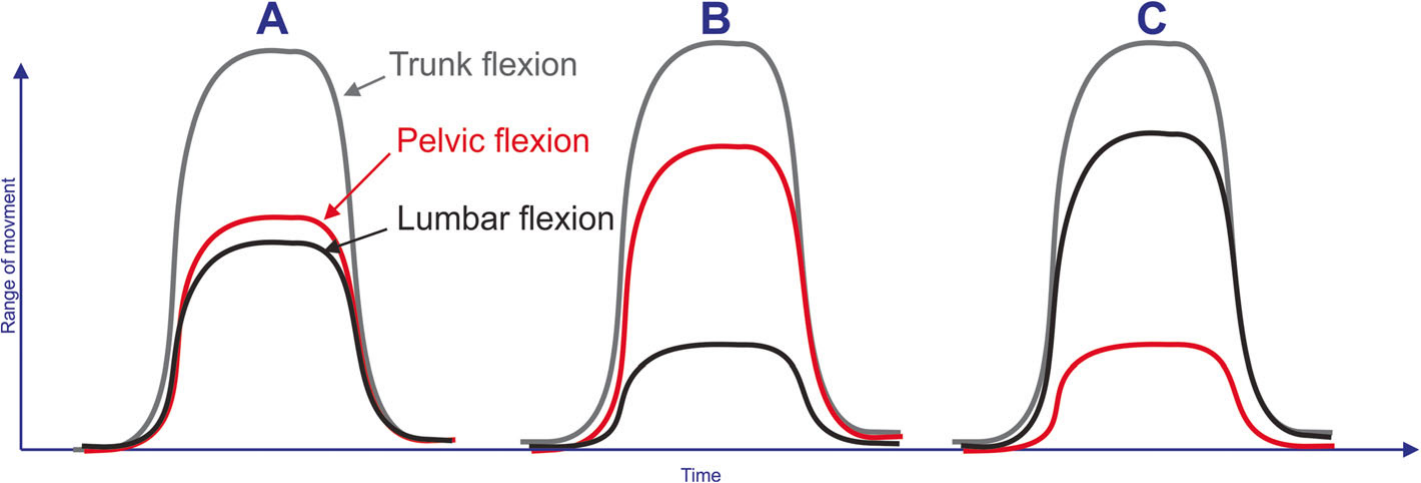
Examples of lumbo-pelvic rhythm movement patterns for flexion. ROM = ROM; Trunk flexion ROM = *grey line*, pelvic (hip) flexion ROM = *red line*, and lumbar flexion ROM = *black line*. Peak trunk flexion angles, recorded with the T12 sensor, consist of two components: (1) pelvic (pelvis-on-hip) movement and (2) lumbar movement. **a** Typical flexion movement pattern of slightly greater pelvic compared to lumbar contribution to trunk movement. **b** Stiff lumbar spine with small lumbar contribution and mostly pelvic movement contributing to trunk flexion. **c** Greater lumbar and relatively smaller pelvic movement

Peak angles were calculated for trunk and pelvic sensors to indicate maximum angular displacement at T12 (trunk movement) and S2 (pelvic/hip movement). Lumbar movement (movement between T12 to S2) was calculated by subtracting pelvic movement (movement of the lower sensor at S2) from trunk movement (movement of the upper sensor at T12). In addition to static posture and ROM, data on ‘lumbar versus pelvic’ contribution to flexion, extension and lateral flexion were collected during each movement. This is shown graphically in Fig. 3. A summary measure of this pattern of lumbar versus pelvic contribution to trunk movement (lumbo-pelvic rhythm) was estimated by calculating the percentage contribution of lumbar ROM to peak trunk ROM for flexion, extension and lateral flexion.

### Statistical analysis

Participant demographics (gender, BMI, pain and activity limitations) were summarised.

### Comparing ROM for participants with and without LBP

Mean ROM scores for each of the repetitions (three tests each of five repetitions) for each movement, for LBP and NoLBP participants, were tested for differences between groups using a repeated measures ANOVA.

### Consistency in repeated measurements

To examine the overall consistency in repeated movements, the standard deviation of all measurements of a movement for each participant was calculated. Differences in standard deviations between groups were tested using independent t-tests.

### Within-test repeated movement consistency

Each of the three tests consisted of five repetitions for each movement. We considered that the best estimate of a person’s ROM would most likely be an average of repeated measurements. Before commencing analysis of the magnitude of error in movement estimates, the five repetitions for Test 1 were examined to determine whether any of the repetitions were systematically different from others. Systematic variation for specific repetitions was assessed using a paired *t*-test to compare the mean for the first repetition to the mean for each of the other repetitions; this was repeated for repetitions 2, 3, 4 and 5, for each movement, and for LBP and NoLBP participants separately. Based on this analysis, we made decisions regarding the repetitions that were suitable for inclusion in subsequent analyses.

### Movement consistency between tests on the same day (inter-rater reliability)

The average of stable repetitions was used as best evidence of the typical movement for each participant. Consistency between repeated tests was estimated using the two-way, random effects, absolute agreement between two raters, Intraclass Correlation Coefficient (ICC 2, 2) statistic. The magnitude of differences between repeated tests was summarised using Bland-Altman plots with 95 % limits of agreement (LOA) and the minimal detectable change (MDC_90_) statistic. These were calculated using the standard deviation of the differences between repeated tests multiplied by 1.65 for the MDC_90_ and 1.96 for 95 % confidence levels (LOA). The MDC_90_ metric with its 90%CI balances statistical rigour with clinical utility in deciphering changes in measurements.

### Movement consistency between tests on different days (7–14 days after the first test: intra-rater reliability)

Methods used to calculate the consistency of measurements taken on the same day were repeated for measurements taken on two test occasions 7–14 days apart. The conceptual framework and definitions of reliability used in this study were those published by the COSMIN group [34]. All analyses were performed using a statistical software package (STATA, version 12).

## Results

### Demographics

Participant gender, age, BMI, LBP intensity and activity limitation are presented in Table 1. There was a significant difference between groups in age. People with LBP were, on average, 10.3 years older than people without LBP.

**Table 1:** Participant demographics

	Number	Gender (% female)	BMI (kg/m^2^)	Age (years)	Pain (0–10 scale)	Activity limitation (RMDQ 24)
NoLBP group	32	42 %	24.4 ± 3.1	35.5^a^ ± 12.4	No pain	No activity limitation
LBP group (Test 1)	30	50 %	24.1 ± 5.6	45.8 ± 11.6	4.5 ± 1.3	6.2 ± 3.5
LBP group (Test 3)					4.5 ± 1.3	4.7 ± 2.6^b^

All numbers indicate mean ± standard deviation

^a^Significant difference in age between groups, *p* = .001

^b^Significant difference for activity limitation between Test 1 versus Test 3 in the LBP group

### Comparing ROM for participants with and without LBP

**Fig. 4:**
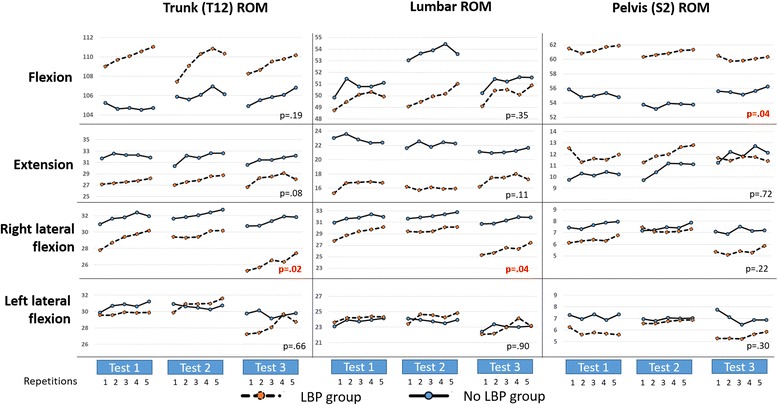
Repetition consistency: total range (trunk ROM) and its components of lumbar ROM and pelvic ROM are presented for LBP and NoLBP participants for measurements taken on each of the three test occasions. P values reflect differences between NoLBP and LBP groups with significance set at > .05. ROM = ROM

Peak ROM scores for movement repetition are illustrated in Fig. 4 and detailed in Table 2. Despite the typical differences in mean scores between LBP and NoLBP participants, these were significant only for pelvic ROM in flexion (LBP 60.8°, NoLBP 54.8°, F(1,63) = 4.31, *p* = 0.04), lumbar ROM in right lateral flexion (LBP 22.2°, NoLBP 24.6° F(1,63) = 4.48, *p* = .04 and trunk ROM in right lateral flexion (LBP 28.4°, NoLBP 31.7°, F(1,63) = 5.9, *p* = .02).

**Table 2:** Lordosis and ROM scores, and consistency between tests (degrees)

Movement	Region	Back pain status	ROM^a^				Inter-rater agreement (same-day, different raters) Test 1 versus Test 2		Intra-rater agreement (different-days, same rater) Test 1 versus Test 3	
			Test 1	Test 2	Test 3	Average for all 3 Tests	Mean & SD of differences between Test 1 & Test 2^b^	Minimal detectable change score	Mean & SD of differences between Test 1 & Test 3^b^	Minimal detectable change score
Lordosis^c^	Lumbar lordosis	NoLBP	−29.6 ± 11.2	−31.2 ± 11.3	−29.4 ± 10.8	−30.1 ± 11.1	1.5 ± 6.9	±11.3	−0.5 ± 9.1	±15.0
		LBP	−27.1 ± 11.6	−28.1 ± 10.5	−28.2 ± 11.8	−27.8 ± 11.2	1.0 ± 5.4	±8.8	0.2 ± 9.0	±14.8
	Trunk angle	NoLBP	−9.9 ± 5.7	−10.5 ± 5.1	−11.0 ± 4.2	−10.4 ± 5.0	0.6 ± 4.2	±6.9	1.2 ± 4.7	±7.7
		LBP	−9.5 ± 5.5	−9.4 ± 4.0	−9.9 ± 4.5	−9.6 ± 4.7	0.0 ± 3.7	±6.1	0.3 ± 3.4	±5.6
	Pelvic angle	NoLBP	19.7 ± 10.0	20.7 ± 9.6	18.4 ± 9.6	19.6 ± 9.7	−1.0 ± 5.5	±9.0	1.7 ± 7.2	±11.9
		LBP	17.6 ± 9.3	18.7 ± 10.8	18.4 ± 10.4	18.2 ± 10.1	−1.1 ± 5.8	±9.6	0.0 ± 7.9	±13.0
Flexion^c^	Trunk (T12) angle	NoLBP	104.9 ± 15.4	106.4 ± 15.5	105.8 ± 15.7	105.7 ± 15.4	−1.5 ± 4.1	±6.8	−0.4 ± 5.7	±9.3
		LBP	110.4 ± 14.3	110.2 ± 13.2	109.6 ± 13.1	110.1 ± 13.4	0.2 ± 5.3	±8.7	−0.4 ± 6.2	±10.2
	Lumbar range	NoLBP	51.2 ± 8.1	54.1 ± 8.9	50.9 ± 10.1	52.1 ± 9.1	−2.9 ± 6.6	±10.8	−0.4 ± 7.9	±13.0
		LBP	49.9 ± 11.6	50.1 ± 11.4	50.5 ± 11.5	50.2 ± 11.3	−0.2 ± 5.0	±8.4	−0.2 ± 8.4	±14.0
	Pelvic (S2) angle	NoLBP	54.9 ± 15.3	53.7 ± 14.6	55.8 ± 15.5	54.8 ± 15.0^d^	1.2 ± 5.0	±8.2	0.2 ± 6.6	±10.9
		LBP	61.0 ± 12.4	60.0 ± 14.4	61.2 ± 12.4	60.8 ± 13.2^d^	0.4 ± 7.1	±11.8	−1.0 ± 9.9	±16.6
Extension^c^	Trunk angle	NoLBP	32.3 ± 8.9	32.3 ± 9.5	31.7 ± 7.3	32.1 ± 8.6	0.0 ± 6.1	±10	−0.6 ± 6.0	±9.9
		LBP	27.1 ± 7.0	26.2 ± 7.6	27.4 ± 6.2	26.9 ± 7.0	−0.9 ± 3.9	±6.3	1.0 ± 4.4	±7.2
	Lumbar range	NoLBP	22.8 ± 13.9	22.3 ± 12.2	21.2 ± 12.4	22.1 ± 12.8	−0.5 ± 7.6	±12.5	−2.2 ± 11.3	±18.6
		LBP	15.1 ± 8.5	15.2 ± 10.6	15.6 ± 7.2	15.2 ± 8.9	0.1 ± 5.5	±9.0	1.4 ± 3.8	±6.2
	Pelvic angle	NoLBP	11.3 ± 8.5	11.5 ± 8.2	12.7 ± 9.3	11.8 ± 8.6	0.2 ± 5.9	±9.7	1.8 ± 8.4	±13.8
		LBP	12.3 ± 8.4	11.3 ± 9.7	12.0 ± 7.8	11.9 ± 8.7	−1.1 ± 6.2	±10.1	−0.3 ± 4.6	±7.6
Left lateral flexion^c^	Trunk angle	NoLBP	31.2 ± 6.6	30.7 ± 6.0	29.9 ± 5.4	−30.6 ± 6	−0.5 ± 4.1	±6.9	−0.8 ± 4.9	±8.1
		LBP	29.8 ± 6.0	31.1 ± 6.5	28.5 ± 6.1	−29.9 ± 6.2	1.3 ± 3.9	±6.5	−0.7 ± 3.4	±5.7
	Lumbar range	NoLBP	24.1 ± 4.7	23.9 ± 4.3	23.3 ± 4.6	−23.8 ± 4.5	−0.2 ± 3.3	±5.5	−0.2 ± 3.9	±6.6
		LBP	24.3 ± 5.3	24.6 ± 5.9	23.1 ± 5.9	−24.1 ± 5.7	0.3 ± 3.5	±5.8	−1.1 ± 3.2	±5.3
	Pelvic angle	NoLBP	7.3 ± 4.1	7.1 ± 3.8	6.9 ± 3.6	−7.4 ± 3.8	−0.2 ± 2.5	±4.2	−0.5 ± 2.7	±4.5
		LBP	5.7 ± 2.8	6.8 ± 3.7	5.5 ± 3.5	−6.0 ± 3.4	1.1 ± 2.6	±4.3	0.3 ± 2.4	±4.1
Right lateral flexion^c^	Trunk angle	NoLBP	31.9 ± 6.0	32.4 ± 6.5	31.6 ± 6.5	32 ± 6.2	−0.5 ± 2.9	±4.9	−0.2 ± 2.7	±4.5
		LBP	29.5 ± 5.1	29.8 ± 5.1	26.5 ± 5.7	28.8 ± 5.4	−0.3 ± 4.0	±6.6	−2.6 ± 3.5	±5.8
	Lumbar range	NoLBP	24.4 ± 4.6	24.9 ± 4.5	24.7 ± 4.6	24.7 ± 4.5	−0.5 ± 2.4	±4.0	−0.7 ± 3.1	±5.2
		LBP	23.2 ± 5.2	22.8 ± 4.6	21.3 ± 5.7	22.3 ± 5.6	0.4 ± 3.0	±5.1	1.9 ± 2.6	±4.3
	Pelvic angle	NoLBP	7.7 ± 3.9	7.7 ± 4.0	7.1 ± 3.8	7.5 ± 3.9	0.0 ± 2.5	±4.1	0.6 ± 2.7	±4.5
		LBP	6.4 ± 2.9	7.1 ± 3.3	5.4 ± 3.3	6.4 ± 3.2	−0.7 ± 3.1	±5.1	0.7 ± 2.8	±4.8

Legend: *LBP* LBP group, *NoLBP* no LBP group, *ROM* ROM

^a^ROM and standard deviation data represent the group mean and standard deviation (SD). The standard deviation indicates the magnitude of differences between individuals within the group

^b^These data are derived from the difference in ROM between tests for each individual, (i.e., Test 1 versus 2, Test 1 versus 3) then calculating group mean and SD of the difference scores

^c^See Table 4 for numbers (n) of participants in each group

^d^Indicates significant difference between groups

### Consistency in repeated measurements

There were no significant differences between LBP and NoLBP participants in consistency of the 15 repetitions of each movement (5 repetitions × 3 tests) with the exception of trunk movement during right lateral flexion, where the standard deviation was significantly greater for the LBP group (2.7° ± .25°) compared to the NoLBP group (1.98° ± .14°).

### Within-test repeated movement consistency

On examination of pairwise comparisons of repetitions 1 to 5, little evidence was found of significant effects attributable to repetition. Exceptions were lumbar flexion, and right lateral flexion (trunk and lumbar ROM) where (typically for both groups) ROM for the first repetition exhibited significantly smaller ROM than all other repetitions. Figure 4 shows similar patterns when other movements were considered. Consequently, repetition one was removed from subsequent analyses.

### Movement consistency between tests

#### Lordosis and ROM

Table 2 summarises data for lordosis and ROM. Mean lordosis angles (across all three tests) for the two groups were not significantly different: 30.1° ± 11.1° for the NoLBP group and 27.8° ± 11.2° for the LBP group. The minimal detectable change based on the middle 90 % of scores (MDC_90_) for measurements of lordosis taken on the same day was ±11.3° for the NoLBP group and ±8.8° for the LBP group, and approximately ±15° (both groups) for different-day comparisons.

**Fig. 5:**
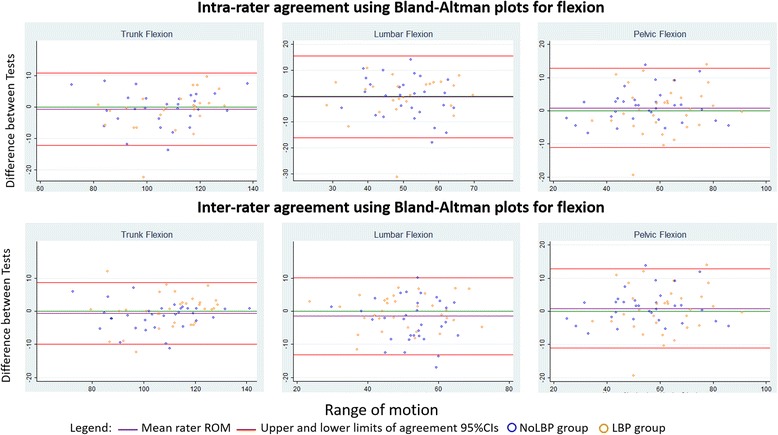
Bland-Altman plots for trunk, lumbar and pelvic lumbar flexion

Different-day measurements generally showed greater inconsistency than measurements taken on the same day. For example, trunk flexion for the LBP group would have to change by more than ± 8.7° (MDC_90_) between tests on the same day for 90 % confidence that observed changes were not due to typical variation in these measurements. This increases to ± 10.2° change for tests on different days. An example of Bland Altman plots displaying the limits of agreement (95 % confidence intervals), for flexion, can be seen in Fig. 5. Trunk ROM measurement consistently showed greater stability compared to lumbar or pelvic ROM measurements for same-day and different-day comparisons. For example, for the LBP group, the MDC_90_ of trunk flexion for different-day tests was 10.2°, compared to an MDC_90_ of 17° for lumbar ROM, and an MDC_90_ of 19° for pelvic ROM.

#### Lumbo-pelvic rhythm

Table 3 summarises the percentage contribution of lumbar ROM to trunk ROM (lumbo-pelvic rhythm). A significant difference between groups was seen for flexion (NoLBP 51.3 % ± 9.4 %, LBP 45.8 % ± 8.6 %, F(1,63) =4.20, *p* = .0445). MDC_90_ scores for lumbo-pelvic rhythm suggest changes of relative lumbar versus pelvic contribution to trunk movement of between 9 and 15% would, for 90 % of tests, indicate true change for flexion and lateral flexion, while changes between 36 and 56 % are required to be similarly sure of true change for extension (see Table 4).

**Table 3:** Lumbo-pelvic rhythm (expressed as the percentage of lumbar contribution to trunk ROM) and consistency between tests

Movement	Back pain status	Average % Lumbar movement for each test^a^				Inter-rater agreement (same-day, different raters)		Intra-rater agreement (different-days, same rater)	
		Test 1	Test 2	Test 3	Average for all 3 Tests	Mean & standard deviation of differences^b^ between Test 1 vs Test 2	Minimal detectable change (MDC_90_)	Mean & standard deviation of differences^b^ between Test 1 vs Test 3	Minimal detectable change (MDC_90_)
Flexion^c^	NoLBP	51.9 % ± 9.6 %	50.0 % ± 9.0 %	52.0 % ± 9.6 %	51.3 % ± 9.4 %	1.9 % ± 5.5 %	9.1 %	0.8 % ± 7.0 %	11.5 %
	LBP	45.4 % ± 8.9 %	45.6 % ± 8.6 %	46.4 %10.7 %	45.8 % ± 8.6 %	0.2 % ± 5.9 %	8.5 %	0.5 % ± 9.4 %	15.5 %
Extension^c^	NoLBP	68.4 % ± 34.0 %	68.9 % ± 31.2 %	66.6 % ± 33.2 %	68.0 % ± 32.3 %	0.5 % ± 22 %	36.3 %	2.6 % ± 34.2 %	56.2 %
	LBP	58.2 % ± 30.2 %	56.2 % ± 30.6 %	59.0 % ± 29.7 %	56.9 % ± 33.7 %	2.0 % ± 25.2 %	41.4 %	1.1 % ± 31.8 %	52.3 %
Left lateral flexion^c^	NoLBP	78.5 % ± 10.0 %	78.7 % ± 9.0 %	79.0 % ± 9.8 %	78.6 % ± 9.5 %	0.2 % ± 7.3 %	12.0 %	0.6 % ± 7.2 %	11.8 %
	LBP	81.6 % ± 8.2 %	79.2 % ± 10.2 %	81.1 % ± 10.4 %	80.6 % ± 9.6 %	2.4 % ± 7.3 %	12.0 %	1.6 % ± 7.2 %	11.8 %
Right lateral flexion^c^	NoLBP	77.4 % ± 9.5 %	78.2 % ± 8.8 %	79.3 % ± 8.3 %	78.0 % ± 9.1 %	0.7 % ± 6.8 %	11.2 %	2.3 % ± 6.3 %	10.4 %
	LBP	78.4 % ± 9.4 %	76.6 % ± 9.4 %	80.2 % ± 11.6 %	78.3 % ± 10.0 %	1.8 % ± 8.2 %	13.5 %	1.0 % ± 8.9 %	14.6 %

^a^Calculated by dividing lumbar ROM over trunk ROM then converting to percentage

^b^See explanation in Table 3 footnote regarding methods used in calculating the SD of difference scores

^c^See Table 4 for numbers (n) of participants in each group

**Table 4:** Inter-rater and Intra-rater reliability (Intraclass Correlation Coefficients using mean of repetitions 2–5)

Interrater	NoLBP subjects				LBP subjects			
	*n* =	T12 angle	Pelvic angle	Lumbar ROM	*n* =	T12 angle	Pelvic angle	Lumbar ROM
Flexion	32	.98 (.96 to .99)	.97 (.94 to .99)	.80 (.56 to .91)	32	.96 (.92 to .98)	.92 (.84 to .96)	.95 (.90 to .98)
Extension	31	.88 (.74 to .94)	.87 (.72 to .93)	.91 (.81 to .96)	28	.95 (.90 to .98)	.77 (.52 to .89)	.94 (.87 to .97)
Lordosis	33	.83 (.65 to .91)	.91 (.83 to .96)	.90 (.79 to .95)	32	.83 (.65 to .92)	.91 (.81 to .96)	.94 (.87 to .97)
Lateral Flexion left	33	.88 (.76 to .94)	.89 (.77 to .94)	.84 (.68 to .92)	32	.89 (.76 to .94)	.79 (.56 to .90)	.89 (.78 to .95)
Lateral flexion right	33	.94 (.88 to .97)	.88 (.72 to .95)	.92 (.84 to .96)	32	.82 (.64 to .91)	.67 (.33 to .83)	.89 (.79 to .95)
Intrarater	NoLBP subjects				LBP subjects			
Flexion	28	.97 (.93 to .99)	.95 (.90 to .98)	.86 (.68 to .94)	25	.95 (.89 to .98)	.86 (.69 to .94)	.86 (.69 to .94)
Extension	28	.84 (.64 to .92)	.71 (.38 to .86)	.79 (.54 to .90)	21	.94 (.88 to .98)	.67 (.25 to .86)	.94 (.87 to .97)
Lordosis	30	.71 (.40 to .86)	.84 (.68 to .93)	.81 (.59 to .91)	25	.89 (.74 to .95)	.82 (.60 to .92)	.85 (.65 to .93)
Lateral Flexion left	30	.77 (.53 to .89)	.85 (.69 to .93)	.76 (.49 to .89)	25	.92 (.82 to .96)	.83 (.61 to .92)	.92 (.81 to .96)
Lateral flexion right	30	.95 (.90 to .98)	.88 (.75 to .94)	.89 (.77 to .95)	25	.85 (.46 to .94)	.70 (.34 to .87)	.92 (.68 to .97)

Legend: ROM = ROM Intraclass correlation co-efficients (ICC 2,2) and 95 % confidence intervals

#### Inter-rater and intra-rater reliability

ICCs (Table 4) across all measured characteristics averaged *r* = .88 (range .80 to .98) for same-day inter-rater reliability and *r* = .85 (range .67 to .97) for different-day intra-rater reliability. All ICCs were below *P* = .005. The results for both intra and inter rater agreement demonstrate good to excellent agreement for almost all comparisons [35].

## Discussion

### Overview

In this study, we assessed people with and without LBP and determined the consistency in measurements of their standing lordosis, active movement range and lumbo-pelvic rhythm over two tests on the same day and a third test 7–14 days later. We found that the LBP and NoLBP participants had similar standing lordosis angles and ROM, with the exception of greater pelvic ROM in flexion (LBP group), and greater trunk and lumbar ROM in right lateral flexion (NoLBP group). Although the LBP group demonstrated similar trunk ROM during flexion, this appeared to have been achieved through relatively greater pelvis/hip contribution. In addition, we found no significant difference in movement consistency between the NoLBP and LBP groups. Lastly, we found good to excellent inter-rater (same day) and intra-rater (different days) reliability for most movements, with MDC_90_ estimates for expected variation between tests in the order of 5–15° and MDC_90_ estimates for lumbar contribution to lumbo-pelvic rhythm in flexion and lateral flexion that ranged from 8 to 15 %. In contrast, the MDC_90_ estimates for lumbar contributions to extension showed an expected variability that was in the order of 36–56 % and these findings may limit the clinical utility of monitoring changes in lumbar contribution to extension.

### ROM and variability comparisons

A recent meta-analysis identified that, on average, people with chronic LBP have less lumbar ROM than people who do not have LBP [22]. Our data did not demonstrate any significant difference between groups in lumbar ROM, although there was a trend towards there being more hip and less lumbar spine involved in achieving flexion ROM for people with LBP (Fig. 4). In addition, we noted less lumbar extension in people with LBP although this also did not achieve significance. These observations warrant confirmation through studies of independent samples of people with and without LBP.

Clinical utility depends on how much change a clinician expects to see and knowledge of how much change is due to biological variation and measurement error. ROM data for all components (i.e., trunk, lumbar and pelvic ROM) of flexion and lateral flexion, and for extension (trunk ROM only) indicate sufficient stability to be potentially clinically useful with MDC_90’s_ of 5–15° (flexion), 4–8° (lateral flexion) and 6–10° (trunk extension) indicating high probability of true change. However, changes to lumbar and pelvic extension were associated with higher retest variations, with MDC_90’s_ of 10–14° (pelvic movements) and up to 19° (lumbar spine movements). These findings may limit the clinical utility of using changes in lumbar spine extension ROM to monitor progress.

Trunk angle measurements were generally associated with smaller retest variations than lumbar or pelvic angle measurements, which may inspire the argument that trunk ROM is the more sensitive and potentially valuable outcome measure. Our data indicate however that people with LBP appear to retain full flexion ROM by increasing pelvic/hip movement while limiting lumbar contribution.

Inter-rater (same day) differences between tests were generally smaller than intra-rater (different day) differences. This is a common finding in reliability studies and is likely to be due to a combination of factors that occur between measurement days, such as normal biological variations, minor variations to experimental conditions and possible environmental factors. We studied intra-rater different-day measurements as this reflects common clinical practice, making the results relevant to clinical decision-making.

### Within-test repeated movement consistency

The first repetition of flexion and right lateral flexion movements was significantly different to subsequent repetitions, with similar, non-statistically significant, patterns seen for other movements (see Fig. 4). As a consequence, we used repetitions 2–5 for analysis of movement consistency. This renders the study results relevant to clinicians who allow clients to practice the test before commencing measurement. The first repetition of a test may be affected by apprehension, uncertainty about what is required, fear of pain, movement stiffness, and distraction or inattention, to name only some of the possible factors that might explain this aspect of our data.

### Lordosis

Lumbar lordosis angles are of clinical interest in assessing spinal alignment and postural archetypes. A wide range of group mean lordosis angles, measured by skin surface techniques, have been reported. A recent review of nine studies reported mean lordosis angles ranging from 23° to 55° [22]. Mean (±SD) standing lordosis, measured in this study, ranged from 27° to 31° ± 11°, without any significant differences between LBP and NoLBP groups. In our data, relatively large variability in standing lordosis angles was seen between tests on both the same day and on different days with MDC_90_ scores ranging from 9° to 11^o^ for tests on the same day and up to 15° for tests on different days. This variability may be a test artifact related to precision in sensor placement or it may be true biological variability. We were very particular in attempting precise sensor repositioning in repeated tests and it is unlikely that greater accuracy in sensor placement would be expected in typical clinical practice.

### Lumbo-pelvic rhythm

Various patterns of lumbo-pelvic movement have been described but few patterns have been measured or reported as outcomes. Clinicians are interested in identifying the contributions to trunk movement from hip movement and lumbar spine movement. It has been proposed that when extremes of lumbar or pelvic contribution to trunk flexion are corrected, associated pain can be reduced [10, 11]. This study showed relatively greater hip compared to lumbar contribution for the LBP group. We speculate that this maybe a compensatory mechanism as a response to reduced lumbar ROM. A recent meta-analysis (six studies) of typical lumbo-pelvic rhythm showed similar but non-significant findings of reduced lumbar contribution to trunk flexion [22]. Although lumbo-pelvic rhythm has been reported using a lumbar/pelvic angle ratio, we consider that percentage lumbar contributions to trunk movement are easier to visualise and circumvent the complexities associated with interpretation of ratios (that can be affected by both the numerator and the denominator). If trunk movement occurs entirely at the lumbar spine, the lumbo-pelvic rhythm will be 100 %, while a person who bends with the pelvis/hips and without lumbar spine movement will score 0 %.

In our data (Table 3), mean lumbar contribution to trunk flexion ranged from 46 % ± 9 % to 51 % ± 9 %. This is closely consistent with Kim et al. [15] who reported similar mean lumbar contributions to trunk flexion of 45 % ± 9 % to 49 % ± 9 %.

**Fig. 6:**
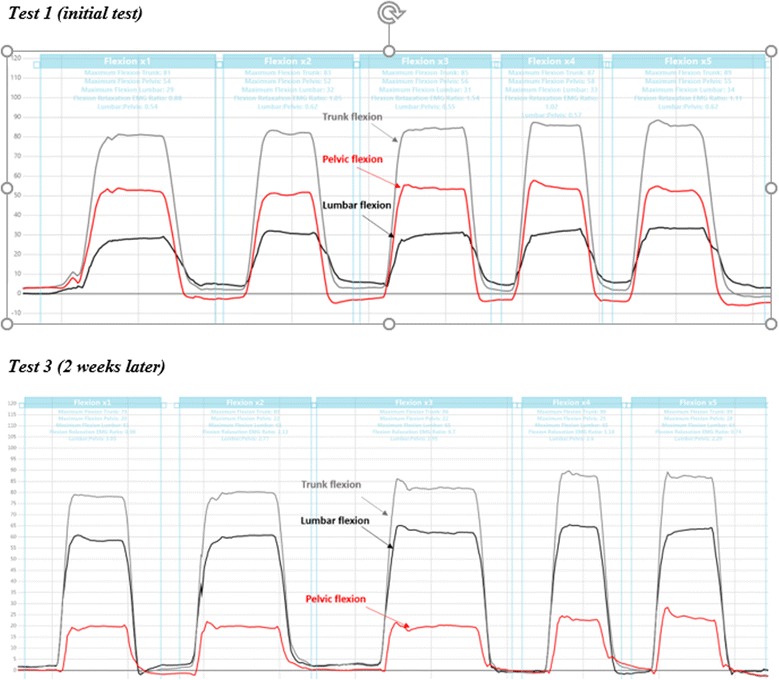
Flexion lumbo-pelvic rhythm differences on Test 1 versus Test 3 (two weeks apart) for participant No. 71 (NoLBP). Illustrates differences between days for flexion and lumbo-pelvic rhythm in one participant. *Grey line* = trunk flexion, *black line* = lumbar flexion and the *red line* = pelvic (hip) flexion

Considerable test-to-test variability in the percentage contribution of pelvic and lumbar movement to trunk flexion was seen in our data for a small number of participants. An example of this variable motor control of lumbar and pelvic movement contribution, while maintaining relatively consistent trunk ROM, is shown in Fig. 6. This NoLBP participant demonstrated an increase in lumbar contribution to trunk flexion from an initial 38 to 74 %, despite little difference in overall trunk ROM of around 80°.

## Limitations of this study

Using a skin surface measurement technique to measure movement has the advantage of being non-invasive and possible within a typical clinical setting. However, any skin surface measurement technique has to be vigilant for artefacts that can occur due to issues such as skin buckling, sensor placement error, loss of sensor adherence to skin, etc. Excessive adipose tissue and skin buckling can alter the orientation of the surface-mounted movement sensor in some people, although simple observation can screen for this type of error. Skin surface measurement also has the inherent issue of sensor placement error, with relatively poor reliability of manual palpation of bony landmarks [30]. However we attempted to reduce this error by additional linear measurement to reduce placement error for subsequent tests.

There is a significant difference between LBP and NoLBP groups for age, with the LBP group being older on average. Other studies have shown that ROM diminishes with age but these changes are more visible in the 5th and 6th decades [36]. While it is possible that age-related differences between groups may account for reduced movement in the LBP group (trunk and lumbar right lateral flexion), it is unlikely age would explain increased ROM (pelvic flexion) or the altered lumbo-pelvic rhythm (where trunk ROM was the similar for both groups).. A significant difference for activity limitation was seen between Test 1 versus Test 3 for the LBP group but the difference between scores was 1.5 on the RMDQ and is unlikely to be clinically meaningful.

Rotational measurements were not technically possible with motion sensors at the time of testing but advances now allow for testing axial rotation. Further research should include rotation.

This study was not powered to test for differences between subgroups within the LBP population (pain intensity, presence of leg pain, mechanism of injury, movement pattern, aggravating activities etc.) so it possible that various subgroup definitions may demonstrate different results.

We conducted multiple ANOVAs when studying the differences in ROM for those with and without LBP, and retained our alpha level at .05 for all comparisons. Some observed differences between groups may therefore be chance findings, and the study findings warrant testing in independent studies.

A further limitation may be the single intra-rater comparison. Further studies could include multiple intra-rater comparisons to increase the robustness of extrapolating these results to other clinicians.

## Conclusion

This study compared the consistency of lumbar lordosis, lumbo-pelvic range of movement (ROM) and lumbo-pelvic rhythm in people with and without low back pain, over three test sessions: two tests on the same day and a third test, 1 to 2 weeks later. There was little difference between the LBP and NoLBP groups for lordosis angle, and most ROM conditions, with the exception of greater pelvic flexion and reduced trunk and lumbar right lateral flexion ROM. Significantly reduced relative lumbar contribution to flexion lumbo-pelvic rhythm was seen in the LBP group. Movement consistency between each test was described by using MDC_90_ to measure between-test differences. Mean lumbar lordosis angles of approximately 30° required around 10° change to have 90 % confidence of seeing true change between same-day tests and 15° for different-day tests. ROM tests showed relatively greater consistency with changes ranging from 5 to15° between tests required to similarly identify true change. Lumbo-pelvic rhythm changes of > 8–15 % lumbar contribution to flexion and lateral flexion trunk ROM indicated probable change, while a larger change of >36–56 % would be needed to be confident of change to an extension lumbo-pelvic rhythm.

## Additional file

Additional file 1: Description and details of measured lumbo-pelvic kinematics. Description of data: Provides information about movements tested, sensor placement and instruction to the subject. (DOCX 12 kb)

## Abbreviations

LBP: Low back pain
NoLBP: Participants without low back pain
RMDQ: Roland Morris Disability Questionnaire
ROM: ROM
