# Supplementary material for: How consistent are lordosis, range of movement and lumbo-pelvic rhythm in people with and without back pain?
Source: BMC Musculoskelet Disord. 2016 Sep 22;17:403. doi: 10.1186/s12891-016-1250-1 (PMC5034504; doi:10.1186/s12891-016-1250-1)
Supplement: Additional file 1: — Description and details of measured lumbo-pelvic kinematics. Description of data: Provides information about movements tested, sensor placement and instruction to the subject. (DOCX 12 kb) [file 12891_2016_1250_MOESM1_ESM.docx]

# Additional File 1

## Description and details of measured lumbo-pelvic kinematics

| **Description** | **Movement characteristics recorded** | **Details** |
| --- | --- | --- |
| Lordosis in neutral defined as usual standing posture | Trunk (T12) and pelvis (S2) angles | Stand for 10 seconds, sensors calibrated relative to vertical |
| Standing pelvic tilt angle and ROM | Angle of pelvis in neutral (pelvis at ‘zero’), full anterior and full posterior tilt | Move from neutral to full anterior tilt, followed by full posterior tilt |
| Flexion ROM (lumbar and hip) | Angle of trunk, pelvis and lumbar spine (T12 angle minus S2 angle), | Move from neutral standing to full flexion, hold for 3 seconds, return to neutral |
| Fixed flexion ROM | Angles of trunk, lumbar spine and pelvis | Move from neutral standing to a fixed height (horizontal chair seat) and return |
| Extension ROM | Angles of trunk, lumbar spine and pelvis | Move from neutral standing to full extension, hold for 3 seconds, return to neutral |
| Lateral flexion ROM | Angles of trunk, pelvis and lumbar spine | Move from neutral (zero) standing to full lateral flexion, hold 3 seconds, return to neutral, repeat bilaterally |

*Legend: ROM= ROM*
